# A 0.6 Mpc H i structure associated with Stephan’s Quintet

**DOI:** 10.1038/s41586-022-05206-x

**Published:** 2022-10-19

**Authors:** C. K. Xu, C. Cheng, P. N. Appleton, P.-A. Duc, Y. Gao, N.-Y. Tang, M. Yun, Y. S. Dai, J.-S. Huang, U. Lisenfeld, F. Renaud

**Affiliations:** 1 Chinese Academy of Sciences South America Center for Astronomy, National Astronomical Observatories, CAS, Beijing, People’s Republic of China; 2 National Astronomical Observatories, Chinese Academy of Sciences (NAOC), Beijing, People’s Republic of China; 3 Caltech/IPAC, MC 6-313, Pasadena, CA USA; 4 Université de Strasbourg, CNRS, Observatoire astronomique de Strasbourg, UMR 7550, Strasbourg, France; 5 Department of Astronomy, Xiamen University, Xiamen, People’s Republic of China; 6 Purple Mountain Observatory & Key Laboratory for Radio Astronomy, Chinese Academy of Sciences, Nanjing, People’s Republic of China; 7 Department of Physics, Anhui Normal University, Wuhu, People’s Republic of China; 8 Department of Astronomy, University of Massachusetts, Amherst, MA USA; 9 Dept. Física Teórica y del Cosmos, Campus de Fuentenueva, Edificio Mecenas, Universidad de Granada, Granada, Spain; 10 Instituto Carlos I de Física Teórica y Computacional, Facultad de Ciencias, Granada, Spain; 11 Department of Astronomy and Theoretical Physics, Lund Observatory, Lund, Sweden

**Keywords:** Galaxies and clusters, Cosmology

## Abstract

Stephan’s Quintet (SQ, co-moving radial distance = 85 ± 6 Mpc, taken from the NASA/IPAC Extragalactic Database (NED)^1^) is unique among compact groups of galaxies^2–12^. Observations have previously shown that interactions between multiple members, including a high-speed intruder galaxy currently colliding into the intragroup medium, have probably generated tidal debris in the form of multiple gaseous and stellar filaments^6,8,13^, the formation of tidal dwarfs^7,14,15^ and intragroup-medium starbursts^16^, as well as widespread intergalactic shocked gas^5,10,11,17^. The details and timing of the interactions and collisions remain poorly understood because of their multiple nature^18,19^. Here we report atomic hydrogen (H i) observations in the vicinity of SQ with a smoothed sensitivity of 1*σ* = 4.2 × 10^16^ cm^−2^ per channel (velocity bin-width Δ*v* = 20 km s^−1^; angular resolution = 4′), which are about two orders of magnitude deeper than previous observations^8,13,20,21^. The data show a large H i structure (with linear scale of around 0.6 Mpc) encompassing an extended source of size approximately 0.4 Mpc associated with the debris field and a curved diffuse feature of length around 0.5 Mpc attached to the south edge of the extended source. The diffuse feature was probably produced by tidal interactions in early stages of the formation of SQ (>1 Gyr ago), although it is not clear how the low-density H i gas (*N*_H i _≲ 10^18^ cm^−2^) can survive the ionization by the intergalactic ultraviolet background on such a long time scale. Our observations require a rethinking of properties of gas in outer parts of galaxy groups and demand complex modelling of different phases of the intragroup medium in simulations of group formation.

## Abstract (web summary)

Atomic hydrogen observations in the vicinity of Stephan’s Quintet are reported, showing a large gaseous structure of around 0.6 Mpc in size in the velocity range of 6,550–6,750 km s^−^^1^.

## Main

**Fig. 1:**
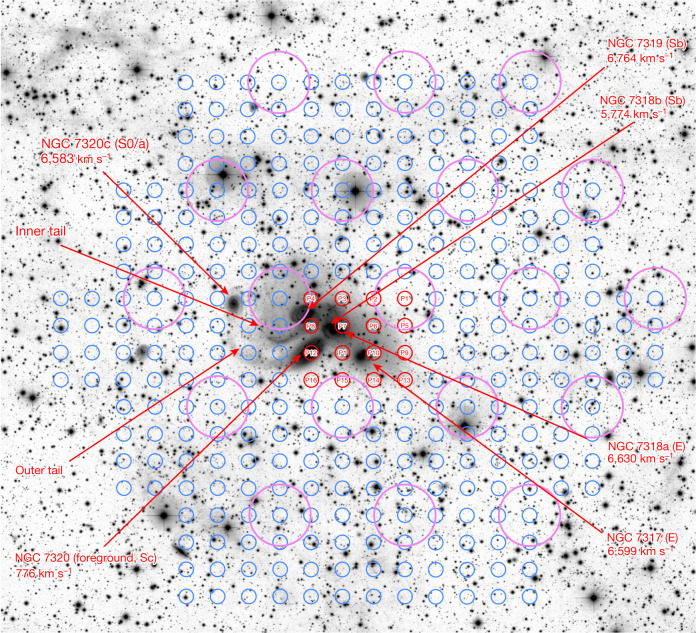
Member galaxies plus main tidal features in SQ and the sky coverage of the FAST observations of the SQ field. The background image is the inverted greyscale map of the deep Canada–France–Hawaii Telescope (CFHT) MegaCam r-band image^12^. The NGC name, radial velocity and the Hubble type are provided for each member galaxy in SQ. The core members include NGC 7317, NGC 7318a and NGC 7319, which are located near the group centre and have similar radial velocities (6,680 ± 85 km s^−1^). NGC 7320c is an ‘old intruder’, which may be responsible for the formation of the inner and outer tails^6,19^. NGC 7318b is a ‘new intruder’, which is currently colliding into the intragroup medium of SQ and triggering a large scale shock^5^. NGC 7320 is a foreground galaxy. The small blue circles mark the positions of individual beams in the H i mapping observations by the FAST 19-beam receiver. The observations were carried out at 16 slightly separated pointings in a 4 × 4 rectangular grid. The small red circles mark the central positions of the FAST 19-beam receiver in these pointings, and the characters P1, P2, …, P16 inside the circles identify the different pointings. The large magenta circles (*D* = 2.9′, that is, the half-power beam size) show the coverage of the 19 beams in the first pointing (see Methods for more details).

**Fig. 2:**
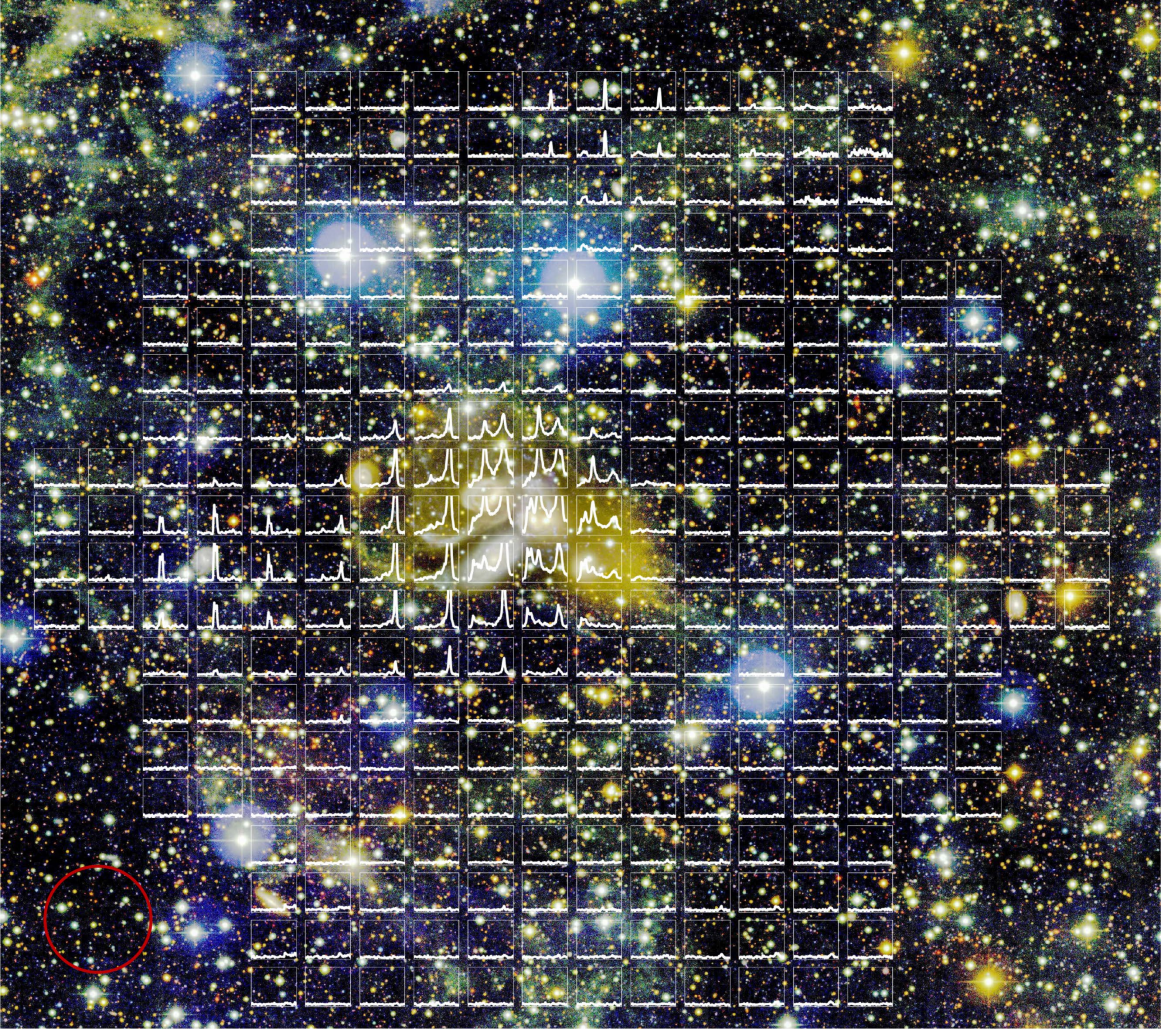
Illustrative plot of the 304 spectra of the H i emission in the SQ field. The underlying optical colour image (u, g, r) is obtained in the deep CFHT MegaCam observation with limiting surface brightness of 29.0, 28.6 and 27.6 mag arcsec^−2^ for the three bands, respectively^12^. The spectra cover the velocity range of 4,600–7,600 km s^−1^ with an average r.m.s. of 0.16 mJy per beam per channel (Δ*v* = 20 km s^−1^). The centre of each spectrum coincides with the pointing position of the FAST beam with which it was obtained. The final data cube includes 304 spectra in 20 km s^−1^ bins. The red circle in the bottom-left corner shows the size of the FAST beam (2.9′, or 72.5 kpc on a linear scale).

Atomic hydrogen (H i) is the least bound component of galaxies and is therefore the easiest (and hence first) to be stripped off and spread around during interactions. Thus, the distribution of the very diffuse H i and its velocity field can provide new information about th earliest interactions. To study the diffuse H i associated with Stephan’s Quintet (SQ), we carried out deep mapping observations of the 21 cm H i emission over a region of around 30′ × 30′ centred on SQ (Fig. 1) using the 19-beam receiver of Five-hundred-meter Aperture Spherical Telescope (FAST). The FAST observations and data reduction are described in the Methods. As shown illustratively in Fig. 2, the final data cube includes 304 spectra in Δ*v* = 20 km s^−1^ channels covering the velocity range of 4,600–7,600 km s^−1^, with an average root-mean-square (r.m.s.) noise of 0.16 mJy per beam and an average beam size of 2.9′. The mapping satisfies the Nyquist sampling criterion with beams separated by 1.4′ in the right ascension direction and 1.2′ in the declination direction. The original data cube has a H i column-density sensitivity of 1*σ* = 1.2 × 10^17^ cm^−2^ per channel, which is improved to 1*σ* = 4.2 × 10^16^ cm^−2^ per channel when smoothed to 4′. Results of the analysis of the whole data cube will be presented elsewhere. In this Article we report only the discovery of a large H i structure in the velocity range of 6,550–6,750 km s^−1^.

**Fig. 3:**
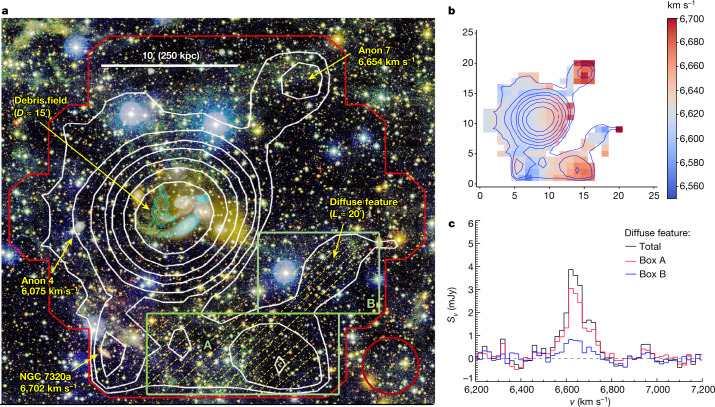
The H i emission in the velocity range of 6,550–6,750 km s^−1^. **a**, Contour map of the integrated H i emission in the velocity range of 6,550–6,750 km s^−1^ overlaid on the composite colour image (u, g, r) of the deep CFHT MegaCam observation. The red circle at the bottom right illustrates the angular resolution (half-power beam size = 4.0′) of the FAST map after smoothing. The contours start from *N*_H i_ = 7.4 × 10^17^ cm^−2^ (at 5.5*σ* level) with an increment of a factor of 2. The red lines delineate the boundary of the FAST observations. The cyan contours in the centre are adopted from the VLA observations for the 6,600 km s^−1^ component of SQ^8^, with angular resolution of 19.4″ × 18.6″. They have the base level at *N*_H i_ = 5.8 × 10^19^ cm^−2^ and the increment of a factor of 2. The area occupied by the newly discovered diffuse feature is filled with a hatch pattern consisting of thin yellow dotted lines. The two green boxes marked by characters A and B cover the diffuse feature. **b**, False colour map of the velocity field of the H i emission in the velocity range of 6,550–6,750 km s^−1^ overlaid by the same contour map shown in **a**. The units of the *x* and *y* axes are in pixels (pixel size: 1.4′ × 1.2′), and the colour of every pixel represents the flux-density weighted mean velocity whose scale is given in the scale bar on the right. **c**, The H i spectrum of the diffuse feature. The red line is the summation of all spectra inside box A and the blue line the summation of those inside box B. The black line is the sum of the two spectra of boxes A and B.

Figure 3a presents the integrated H i emission map in the velocity range of 6,550–6,750 km s^−1^ overlaid on the deep MegaCam optical colour image^12^. The map has an angular resolution of 4.0′ and Δ*v* = 200 km s^−1^ (ten times the channel width) with an H i column-density error of 1*σ* = 1.34 × 10^17^ cm^−2^. The base contour starts from *N*_H i_ = 7.4 × 10^17^ cm^−2^ (at a 5.5*σ* level). The map shows a large H i structure of around 0.6 Mpc in size, which has two parts: an extended source centred on SQ and a diffuse feature attached to the south edge of the source. The extended source encompasses the previously detected 6,600 km s^−1^ H i component associated with the debris field^8,13^. As shown by the cyan contours in Fig. 3a, the high-resolution (beam = 19.4″ × 18.6″) Very Large Array (VLA) observations^8^ detected only the high- density part of the 6,600 km s^−1^ component (*N*_H i_ ≥ 5.8 × 10^19^ cm^−2^), which is confined to the central region (*D* ≈ 0.1 Mpc) of the extended source. Most of the high-density H i gas traces the optically detected inner and outer tails^6^ plus a compact cloud (north-west of NGC 7319) coincident with the intragroup-medium starburst SQ-A^16^. Single-dish H i mapping observations by the Arecibo Telescope and the Green Bank Telescope, which detected lower density H i gas at *N*_H i_ ≈ 5 × 10^18^ cm^−2^ albeit with lower angular resolutions (>3′), have found evidence for this component to be extended on a scale of around 0.2 Mpc (refs. ^20,21^). Our deeper FAST map shows an even larger size with a diameter of around 0.4 Mpc. The diffuse feature has a characteristic column density of approximately 7 × 10^17^ cm^−2^ in an elongated and curved structure of around 0.5 Mpc in length. It appears at the bottom of the FAST mapping and therefore may well reach beyond the map. The faint optical halo (the yellowish diffuse light around SQ in the optical colour image) discovered previously^12^ lies inside the extended source and has no spatial overlap with the newly discovered diffuse feature. The first moment map in Fig. 3b shows that in the velocity field the diffuse feature is linked smoothly with the extended source. The two green boxes marked by characters A and B in Fig. 3a cover the entire diffuse H i feature. The sum of all spectra in these two boxes provides a good measure of the spectrum of the diffuse feature, which is presented in Fig. 3c. The spectrum has a flux-density-weighted mean velocity of 6,633 km s^−1^ and a rather narrow line width of Δ*V*_20_ = 160 km s^−1^ (*ΔV*_20_ is the line width measured at 20% of the peak). The integrated flux is 0.42 ± 0.03 Jy km s^−1^, corresponding to an H i mass of (7.1 ± 0.5) × 10^8^*M*_⨀_, which is only around 3% of the total H i mass of SQ (2.45 × 10^10^*M*_⨀_)^21^. It is worth noting that, although the Green Bank Telescope mapping observations found 65% more H i than the VLA observations, SQ is still slightly deficient in H i abundance compared with normal galaxies (by a factor of around 1.3)^4,21^. The very diffuse H i (*N*_H i_ < 3 × 10^18^ cm^−2^) discovered in this work does not change this H i deficiency significantly.

Two new detections of unresolved sources can also be found in Fig. 3a. NGC 7320a, detected with the signal-to-noise ratio S/N = 36, has an H i mass of *M*_H i_ = (6.3 ± 0.2) × 10^8^*M*_⨀_ and a *v*_H i_ = 6,702 ± 24 km s^−1^. The other source Anon 7, a 4.4*σ* detection, has an H i velocity of 6,654 ± 16 km s^−1^ and an H i mass of (2.2 ± 0.5) × 10^8^*M*_⨀_. More discussions about these two sources are given in the Methods.

**Fig. 4:**
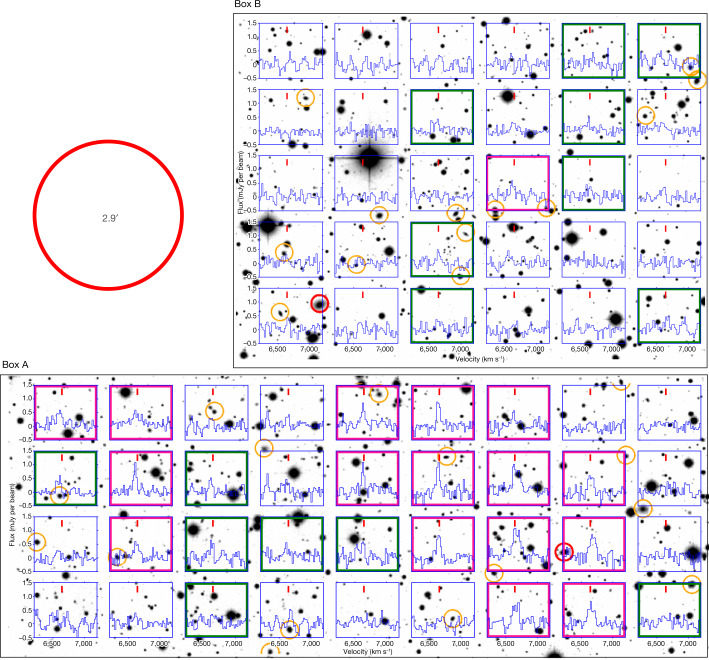
Individual spectra in the diffuse feature region. Individual spectra in box A and box B in Fig. 3a are overlaid on the inverted greyscale map of the CFHT MegaCam r-band image. The centre of each spectrum coincides with the pointing position of the FAST beam with which it was obtained. All spectra are plotted with the same velocity range of [6,200, 7,200] km s^−1^ and flux-density range of [−0.5, 1.5] mJy per beam. The short vertical red line on the top of every spectrum marks the position of *v* = 6,642 km s^−1^ (the flux-density-weighted mean velocity of the diffuse feature). The spectra with detections of S/N > 4 are identified by pink boxes and those with detections of 3 < S/N ≤ 4 by green boxes. Galaxies brighter than *r* = 20 mag are marked by red circles if they have photo-*z* < 0.1 or orange circles if they have photo-*z* ≥ 0.1. The large red circle in the upper-left corner (outside the boxes) illustrates the half-power beam size of the FAST observations.

We examine in Fig. 4 the individual spectra in box A and box B to investigate the physical nature of the diffuse feature. No diffuse stellar radiation is detected in these regions down to the limit of the deep MegaCam image. Spectra of beams with detections of S/N > 4 are marked by pink boxes and those with 3 < S/N ≤ 4 by green boxes. Galaxies brighter than the r-band magnitude *r* = 20 mag found in the SDSS photometric redshift (photo-*z*) catalogue^22^ are also marked in Fig. 4. Only two of them have photo-z < 0.1 (marked by red circles) and the remaining 28 have photo-*z* ≥ 0.1 (orange circles). Given the 1*σ* error of the photo-*z* (δ*z*/(1 + *z*) = 0.02)^22^ and the redshift of SQ (*z* = 0.02), galaxies with photo-*z* ≥ 0.1 are very unlikely to be at the same redshift of SQ (probability < 0.001). We can rule out with high confidence the possibility of the diffuse feature being associated with a collection of gas-rich galaxies (even including those fainter than *r* = 20 mag), because it needs at least four such galaxies to cover all beams with significant detections of S/N > 4 (one for those in the upper-left corner of box A, two for those in the right half of box A and one for that in box B) and the probability that they happen to have about the same radial velocity is extremely low.

H i clouds without stellar counterparts have been found in and around many galaxy groups/clusters^23–26^. Most of them, as the authors of ref. ^27^ have argued, can be explained by tidal debris of galaxy interaction involving very extended H i disks instead of ‘dark’ or ‘almost dark’ galaxies. Given its location and velocity, the diffuse feature is most likely to be related to the debris field. A hypothetical scenario for the formation of the diffuse feature is that NGC 7320a (*v* = 6,702 km s^−1^ and currently around 300 kpc away from the SQ centre) passed through the SQ centre approximately 1.5 Gyr ago (assuming a relative transverse velocity of 200 km s^−1^) and pulled out from one of the core member galaxies of SQ a tidal tail, which developed into the diffuse feature we see now. Another possibility is that, like the large Leo Ring (*D* ≈ 0.25 Mpc)^28^, the diffuse feature could be the product of a high-speed head-on collision between another old intruder and one of the core members of SQ. In this scenario, the collision triggers an expanding density wave that pushes gas in an extended H i disk of the target galaxy outwards to form a very large ring, of which the diffuse feature is the high-density part. A candidate for such an intruder could be Anon 4 (*v* = 6,057 km s^−1^, *M*_H i_ = 1.1 × 10^9^*M*_⨀_)^8^, which spatially coincides with optical galaxy LEDA 141041 (B band magnitude *B* = 18.4 mag). It has a relative radial velocity of around 600 km s^−1^ and a projected distance of approximately 0.2 Mpc from the SQ centre. If the relative transverse velocity is around 200 km s^−1^, it would have taken approximately 1 Gyr for Anon 4 to move to the current position after the collision. Both scenarios proposed above suggest a formation time of the diffuse feature of more than 1 Gyr ago. They are both based on analogies to cases studied in simulations in the literature, which demonstrate that diffuse H i features without a stellar component can be produced in galaxy–galaxy interations^27,28^. However, two questions remain to be answered: (1) Can the tidal feature in either of the scenarios survive the subsequent interactions that triggered the formation of the inner and outer tails of SQ about (3–8) × 10^8^ years ago^18,19^? (2) Can H i structures with column density as low as *N*_H i_ ≲ 10^18^ cm^−2^ exist on timescales of around 1 Gyr? These questions can only be answered by more sophisticated models that are built upon the existing simulations for the formation and evolution of SQ^18,19^. It has been argued that cold gas of *N*_H i_ ≤ 2 × 10^19^ cm^−2^ cannot stay neutral in the intergalactic ultraviolet background radiation for more than 500 Myr (refs. ^21,29^). A plausible solution for this problem is the physical mechanism involving the transition between ionized and neutral phases due to thermal instabilities in the low-density gas^30,31^. New simulations, which are beyond the scope of this Article, shall explore this mechanism.

## Methods

### FAST observations

The deep H i mapping observations were carried out in September and October of 2021 using the FAST 19-beam receiver in the standard ON–OFF mode with the total observation time of 22.4 h including overheads (Extended Data Table 1). The FAST 19-beam L-band Array is currently the largest multibeam feed array for H i observations in the world. Details about its properties and performance can be found in ref. ^32^. The 19 beams are arranged in a hexagonal configuration with the neighbouring beams separated by 5.7′. The observations have a central frequency of 1,391.64 MHz and a frequency coverage of 1,050–1,450 MHz with a resolution of 7.63 kHz (Δ*v* = 1.65 km s^−1^). For the 19 beams, the average half-power beam-width at 1,391 MHz is 2.9′ (Extended Data Table 2). To meet the Nyquist sampling criterion and fill the gaps between beams in the focal plane, we carried out 16 pointings in a 4 × 4 rectangular grid in the north-up orientation (Fig. 1). The final mapping covers a region of around 30′ × 30′ centred on SQ with 304 sky pixels (beam positions in the sky), and the separation between the nearest pixels is 1.4′ in the right ascension direction and 1.2′ in the declination direction. The 1*σ* pointing error of individual beams is 7.9′′ (ref. ^32^). At each pointing, six cycles of ON–OFF integrations were conducted, with the OFF position at 40′ southeast from the ON position. Each ON or OFF took 300 s integration with a sampling frequency of 1 Hz. The total on-target time for each pixel was 1,800 s (Extended Data Table 1). To minimize the effects of standing waves and sidelobes, all observations were confined within the zenith angle of 20°.

Compared with the scan-mapping mode, which is more suitable for large sky surveys such as the FAST Extragalactic HI Survey^33^, our observational strategy provides an alternative for deep and small maps (≤30′), which can take advantage of the ON–OFF mode in more accurately removing various systematic effects such as the standing waves and baseline wobbling.

### Data reduction and calibration

**Extended Data Fig. 1:**
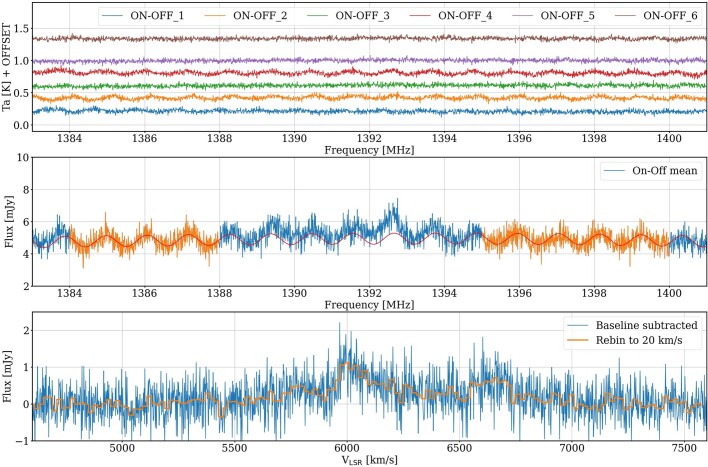
Examples of intermediate products of spectral data reduction. **Upper Panel:** Individual ON-OFF spectra obtained by beam M01 in Pointing 1 observation (i.e. the data obtained at the pixel P1 in Figure 1). An offset of 0.2 K is added to every spectrum relative to the previous one in order to make them separated from each other. **Middle Panel**: The mean of the ON-OFF spectra in the upper panel. The units of the flux density are converted from K to mJy using the gain factor taken from Extended Data Table 2. The red line shows the baseline model derived by fitting the parts of the spectrum free of signals (marked by orange color) using a sinusoidal (representing standing waves) plus a polynomial (for baseline gradient). **Bottom Panel**: The spectrum after the baseline removal. The frequency is converted to radial velocity in the optical convention relative to the local standard rest reference frame (LSR). The orange line presents the rebinned spectrum with the velocity bin-width of 20 km s^−1^.

For each sky pixel observed by a given beam, we reduced the spectral data following a similar procedure to that presented in ref. ^34^. The spectra of the two polarizations were reduced separately (and eventually combined after the consistency check). The data were grouped in ON–OFF cycles. Each ON (or OFF) has 300 samplings which were averaged and calibrated (that is, converted from digital counts to kelvin), resulting in a single raw spectrum. During the observations, a calibration signal of 10 K was injected for a duration of 20 s at the beginning of every ON–OFF cycle, and these data were used to calibrate the antenna temperature *T*_a_. The calibration error is on the order of 10%. Repeating this, we obtained a raw spectrum for every ON or OFF. As examples, the upper panel of Extended Data Fig. 1 presents the individual raw ON–OFF spectra obtained by the M01 beam during the first pointing observation (the P1 pixel in Fig. 1). The mean of these spectra is presented in the middle panel of Extended Data Fig. 1. It is affected significantly by the standing waves, which can be well fitted by a sine function locally. The spectrum has been converted from *T*_a_ (in K) to flux density (in mJy). The gain factor that converts *T*_a_ to flux density (in the units of K Jy^−1^) depends on the frequency and varies from beam to beam. The values for individual beams at 1,391 MHz, derived by interpolating values at other frequencies adopted from ref. ^32^, are presented in Extended Data Table 2. The next step was to remove the standing waves together with the baseline from the spectrum. The bottom panel of Extended Data Fig. 1 presents the final spectrum at sky pixel P1 after the subtraction of a baseline modelled by a sinusoidal (representing the standing waves) plus a polynomial (for the baseline gradient). We converted the frequency to velocity by adopting the optical redshift convention and the local standard of rest reference frame and rebinned the spectrum into bins of Δ*v* = 20 km s^−1^. The above process was carried out repeatedly for every sky pixel observed in our observations.

It is worth noting that, if the standing waves and baseline vary significantly during a given observation, it is better to carry out the removal of the standing waves and baseline for spectra of individual ON–OFFs instead of doing it for their mean. However, in a test in which we did the standing waves and baseline subtraction for each ON–OFF and then used the median of the baseline-subtracted spectra of ON–OFFs as the final spectrum at a given pixel, we got a noisier product. It seems that the wavelength of the standing waves (in the frequency domain) in a given observation is rather constant (although the phase changes from cycle to cycle). Consequently, the effect of the standing waves in the mean of the spectra of individual ON–OFFs is still a well-defined sinusoidal with the same wavelength, which can be easily removed. Hence, because the mean spectrum is less noisy than individual spectra and therefore a more accurate model for the standing waves and the baseline can be obtained, subtracting the baseline model from the mean spectrum can achieve a better result.

The final data cube was constructed from the 304 individual spectra obtained using this technique, with a velocity coverage of 4,600–7,600 km s^−1^ in 20 km s^−1^ bins. A uniform half-power beam-width of 2.9′ was adopted for all spectra, neglecting the small variation of the beam size among different beams.

The actual half-power beam-width of individual beams at 1,391 MHz, derived from interpolations of values adopted from ref. ^2^, are listed in Extended Data Table 2. The units of the flux density of the spectra in the cube are mJy per beam. To find the flux density in units of mJy for a given spectrum, a factor of *A* = (*B*/2.9′)^2^ should be multiplied to the value taken from the cube, where *B* is the half-power beam-width (in arcmin) of the beam with which the spectrum was observed.

**Extended Data Fig. 2:**
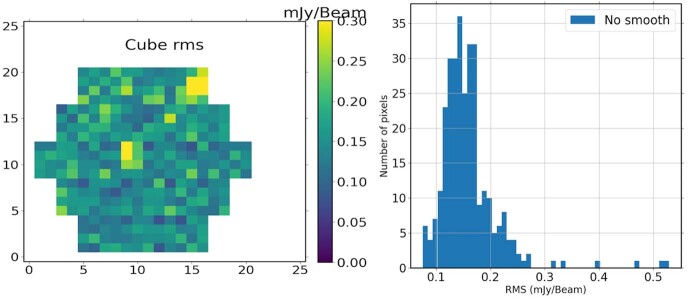
Spectral R.M.S. noise of individual sky pixels. **Left:** The false color image of the r.m.s. noise at different sky pixels. **Right**: Histogram of the distribution of the r.m.s. noise.

In the 16-pointing observations, each of the 19 beams covered 16 adjacent sky pixels. The mean and the standard deviation of the measured r.m.s. noise of these 16 spectra are also listed in Extended Data Table 2. The r.m.s. noise of each spectrum was measured in the two velocity intervals of 4,700–5,000 km s^−1^ and 7,000–7,500 km s^−1^, where no H i signal was detected. Beam M16 stands out as the noisiest beam in the array, with a mean r.m.s. of 0.26 mJy per beam and a standard deviation of 0.14 mJy beam. The false colour map of the r.m.s. noise in the left panel of Extended Data Fig. 2 shows that indeed pixels in the top-right corner that were covered by beam M16 have higher noise than others. The histogram of the distribution of the r.m.s. noise at all sky pixels is shown in the right panel of Extended Data Fig. 2. The mean of the r.m.s. is 0.16 mJy per beam with a standard deviation of 0.05 mJy per beam. It is worth noting that strong radio frequency interference (RFI) in the frequency range of our observations was a serious issue in the early stages of this project. The operation team of FAST did excellent work in discovering and removing the source of the RFI in a relatively short time. All of our observations were carried out after the removal of the RFI source. Consequently, our observations were not affected by any significant RFI.

### Smoothing

**Extended Data Fig. 3:**
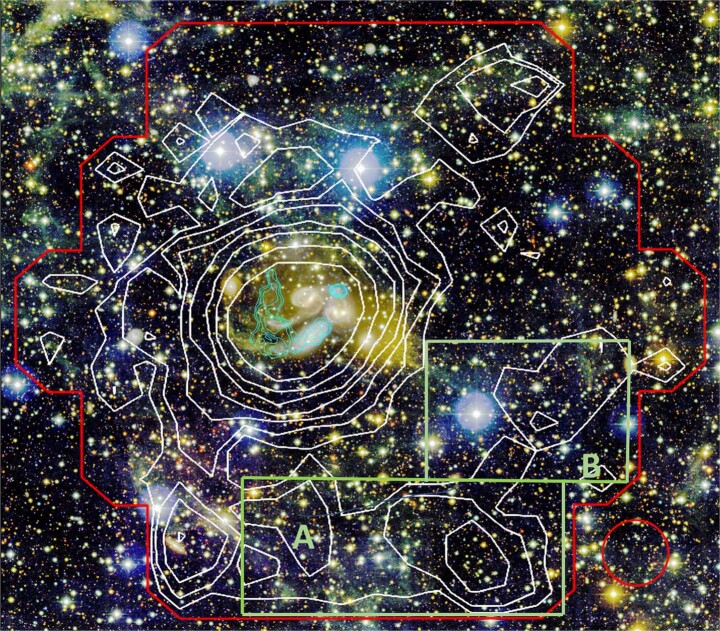
The HI emission in the velocity range of 6550–6750 km s^−1^ (unsmoothed). Contour map of integrated HI emission (unsmoothed) in the velocity range of 6550–6750 km s^−1^ overlaid on the composite color image (u, g, r) of the deep CFHT MegaCam observation. The red circle at bottom-right illustrates the angular resolution (FWHM =2.9') of the FAST map (unsmoothed). The contours start from N_HI_ = 7.4 × 10^17^ cm^−2^ (at 2-σ level) with an increment of a factor of 2. The red lines delineate the boundary of the FAST observations. The cyan contours in the center are adopted from the VLA observations for the 6600 component of SQ^8^, with angular resolution of 19.4"×18.6". They have the base level at N_HI_ = 5.8 × 10^19^ cm^−2^ and the increment of a factor of 2. The same Box A and Box B that mark the location of the diffuse feature in Figure 3a are plotted here to facilitate the comparison between the two figures.

The H i data cube obtained above is highly redundant in the sense that a sky area of the size of a single beam (*D* = 2.9') is covered by multiple beams (beam separation 1.4' x 1.2'). When making channel maps and integrated emission maps from the data cube, applying a Gaussian-kernel convolution (that is, smoothing) makes good use of this redundancy. This minimizes the noise due to the signal fluctuations in adjacent beams and results in significant improvement in the H i column-density sensitivity. The only disadvantage is a slight degradation in the angular resolution. For single-channel maps, the mean r.m.s. of 0.16 mJy per beam corresponds to a H i column-density sensitivity of 1*σ* = 1.2 × 10^17^ cm^−2^ per channel (Δ*v* = 20 km s^−1^). When a smoothing with a Gaussian kernel of full-width at half-maximum (FWHM) = 2.8′ is applied, the H i column-density sensitivity is improved by a factor of 2.9 to 1*σ* = 4.2 × 10^16^ cm^−2^ per channel whereas the angular resolution is degraded only slightly (by a factor 1.4) to 4.0′. The improvement in the H i column-density sensitivity is particularly important for the exploration of diffuse extended emission. In Extended Data Fig. 3 we present the contour map of the integrated H i emission in the velocity range of 6,550–6,750 km s^−1^ before the smoothing. Compared with the map after the smoothing (Fig. 3a), the low H i column-density features in Extended Data Fig. 3 are more fragmented mainly because of the signal fluctuations in adjacent beams. On a linear scale the pre- and after-smoothing resolutions are 72.5 kpc and 100 kpc, respectively, which is not a significant difference given that we are searching for extended diffuse H i gas on a linear scale of a few 100 kpc.

### Sidelobe correction

The data cube is corrected for the sidelobes using the images of individual beams of the 19-beam receiver^32^, which provide information about the point spread functions of the beams. For each beam, a ‘sidelobe responsivity function’ is defined by the difference between the point spread function and the ‘main beam’, the latter being approximated by a two-dimensional Gaussian with the FWHM equal to the half-power beam-width. In the calculation of the sidelobe corrections, we consider only the effects due to the central extended source associated with the SQ group. The FAST observations also detected numerous other H i sources in the SQ neighbourhood in the velocity range of 5,500–7,000 km s^−1^. They are much fainter than the central SQ source and therefore their contributions to the sidelobes are neglected.

**Extended Data Fig. 4:**
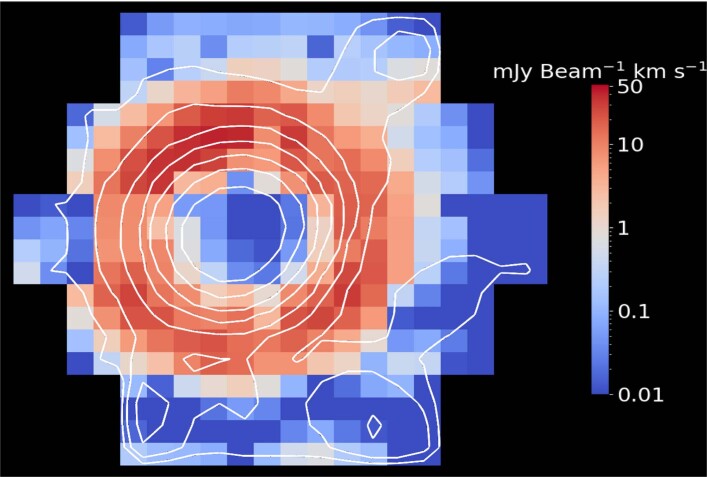
Sidelobe contribution to the integrated HI emission in the velocity range of 6550–6750 km s^−1^. False color image of the sidelobe contribution overlaid by the contour map of integrated HI emission in the velocity range of 6550–6750 km s^−1^ (before the sidelobe correction and after the convolution by a Gaussian kernel of FWHM = 2.8'). The contours start from 20 mJy km s^−1^ beam^−1^ (corresponding to N_HI_ = 7.4 × 10^17^ cm^−2^ for the original beam of FWHM = 2.9') with an increment of a factor of 2.

The first step is to estimate the sidelobe contribution to the map of integrated H i emission in the velocity range of 6,550–6,750 km s^−1^, which encompasses the peak of the H i spectrum of SQ^21^. The original integrated H i emission map (the observed map) is first deconvolved with the main beam of M01 (the central beam of the 19-beam receiver). Then all pixels outside a circular aperture of *D* = 10.6′, within which the central extended source is located, are masked. The result is then taken as our approximation for the ‘truth map’. The sidelobe contribution to any given pixel in the observed map is estimated by the following equation: *F*_sidelobe_ (*x*_*i*_, *y*_*j*_) = ∑_*m*_∑_*n*_*T*(*x*_*m*_, *y*_*n*_) × *R*_*k*_ (*x*_m _− *x*_*i*_, *y*_n  _− *y*_*j*_), where *x*_*i*_ and *y*_*j*_ are the coordinates of the pixel centre, *T*(*x*_*m*_, *y*_*n*_) is the flux in the truth map in the pixel at *x*_*m*_ and *y*_*n*_, *R*_*k*_ is the sidelobe responsivity function of the beam that is pointed at the pixel (*x*_*i*_, *y*_*j*_), and ∑_*m*_ and ∑_*n*_ are summations along the *x* and *y* directions, respectively. Extended Data Figure 4 presents the map of the sidelobe contribution estimated using this method overlaid by contours of the map of integrated H i emission in the velocity range of 6,550–6,750 km s^−1^ (without the sidelobe correction, smoothed by a Gaussian kernel of FWHM = 2.8′). It shows that the sidelobes contribute significantly at the edge of the debris field but have a minimal effect on the diffuse feature in the south.

Neglecting the frequency dependencies of the shapes of both the sidelobes and the central SQ source, we estimate the sidelobe contribution to each channel in the data cube by scaling the map in Extended Data Fig. 4 with a factor of *C*_*v*_ = *S*_*v*_ /*S*_6,550–6,750_, where *S*_*v*_ (in mJy) is the flux density of SQ in the given channel and *S*_6,550–6,750_ (in mJy km s^−1^) is the integrated flux of SQ in the velocity range of 6,550–6,750 km s^−1^. Finally, we obtain the sidelobe corrected data cube by subtracting the estimated sidelobe contribution from every channel map in the cube. The 304 spectra in the resulting data cube are presented illustratively in Fig. 2.

### Detections of two new H i sources in the SQ neighbourhood

**Extended Data Fig. 5:**
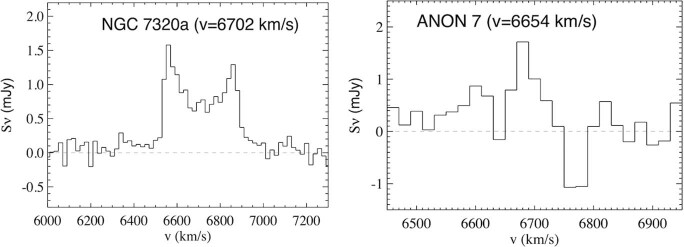
HI spectra of NGC 7320a and Anon 7. HI spectra of two newly detected HI sources (NGC 7320a and Anon 7) in the SQ neighborhood.

**Extended Data Fig. 6:**
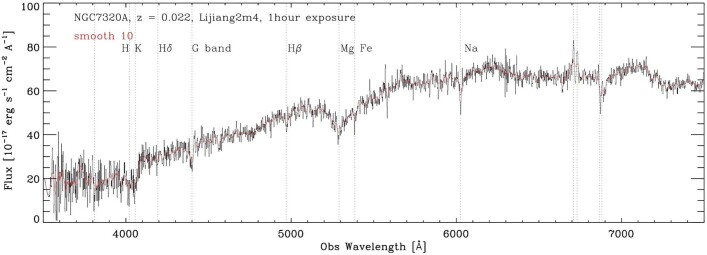
Optical spectrum of NGC 7320a. Optical spectrum of NGC 7320a obtained at Lijiang 2.4 meter telescope. The vertical dotted lines mark the positions of corresponding emission/absorption lines redshifted according to the best-fit redshift z = 0.02243 (v = 6729 km s^−1^).

In the velocity range of 6,550–6,750 km s^−1^ we detected two new unresolved H i sources in the SQ neighbourhood (Extended Data Table 3). Their H i spectra are presented in Extended Data Fig. 5. The spectrum of the source associated with NGC 7320a shows a typical double-horn profile consistent with the highly edge-on optical morphology of the galaxy. To confirm the association of the H i source and the optical galaxy, we made a long-slit optical spectroscopic observation (1 h exposure) for NGC 7320a on the night of 21 December 2021 using the 2.4 m telescope at Lijiang Observatory. The optical spectrum is presented in Extended Data Fig. 6. The radial velocity obtained from the optical spectrum is 6,729 ± 59 km s^−1^, which is consistent with the H i velocity (6,702 ± 24 km s^−1^). The other source is a 4.4*σ* detection without obvious optical counterpart and therefore we name it Anon 7 following the convention in the literature^8,13^.

## Online content

Any methods, additional references, Nature Research reporting summaries, source data, extended data, supplementary information, acknowledgements, peer review information; details of author contributions and competing interests; and statements of data and code availability are available at 10.1038/s41586-022-05206-x.

## Figures and Tables

**Extended Data Table 1:**
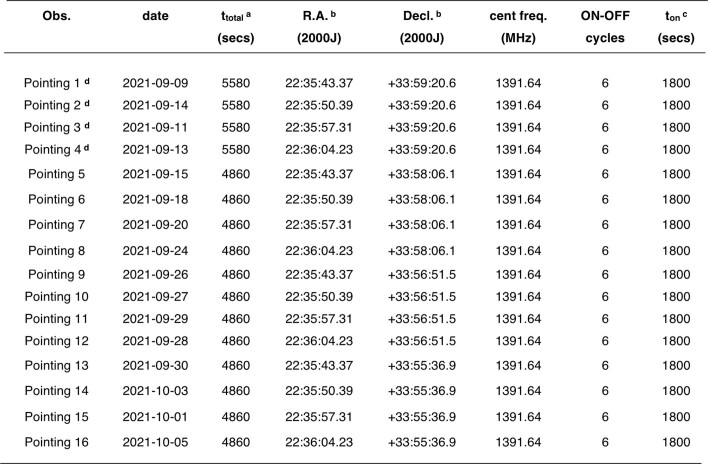
FAST Observations

**a**: Total observation time including all overheads.

**b**: Coordinates of the pointing position of the central beam M01.

**c**: Total on-target time.

**d**: These earlier observations have higher overheads than the later observations.

**Extended Data Table 2:**
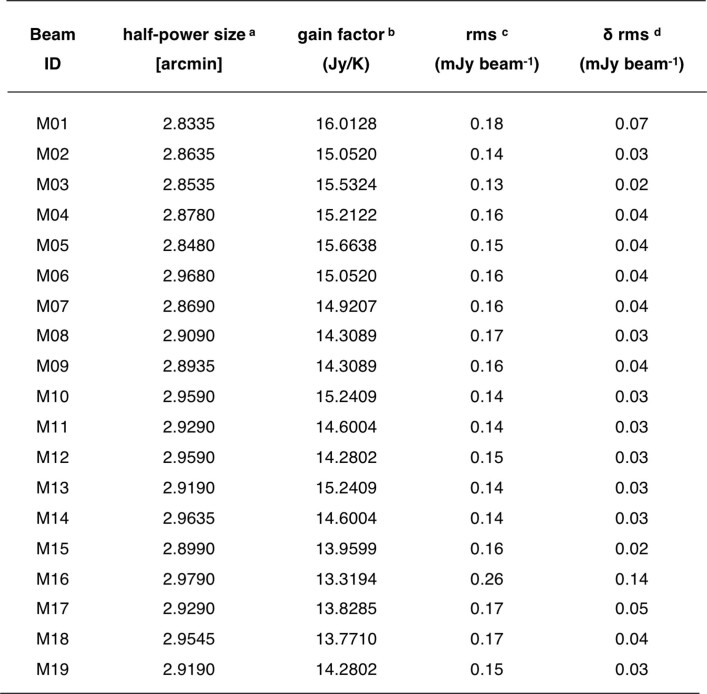
Properties of beams in the 19-beam receiver

**a**: Half-power beam size at 1391 MHz.

**b**: Gain factor at 1391 MHz.

**c**: Mean r.m.s. of the 16 spectra (bin-width of 20 km s^−1^) obtained with the  beam.

**d**: Standard deviation of the r.m.s.

**Extended Data Table 3:**
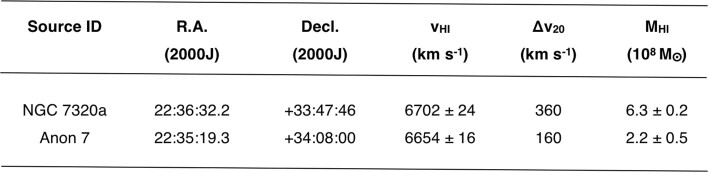
Two new HI sources in the SQ neighborhood

## Data Availability

Observational data are available from the FAST archive (http://fast.bao.ac.cn) 1 year after data collection, following FAST data policy. The data that support the findings of this study are openly available in Science Data Bank at https://www.scidb.cn/s/jiIfee.

## Code availability

The Python and IDL code for the data reduction pipeline is available at https://www.scidb.cn/s/jiIfee.

## References

[1] *NASA/IPAC Extragalactic Database (NED)* (NASA, 2022); https://ned.ipac.caltech.edu.

[2] Stephan ME (1877). Nébuleuses nouvelles, découvertes et observées à l'Obsérvatoire de Marseille. C. r. hebd. séances l'Acad. sci..

[3] Hickson P (1982). Systematic properties of compact groups of galaxies. Astrophys. J..

[4] Verdes-Montenegro L (2001). Where is the neutral atomic gas in Hickson groups?. Astron. Astrophs..

[5] Allen RJ, Hartsuiker JW (1972). Radio continuum emission at 21 cm near Stephan's Quintet. Nature.

[6] Sulentic JW (2001). A multiwavelength study of Stephan's Quintet. Astron. J..

[7] Gallagher SC (2001). Hubble Space Telescope images of Stephan's Quintet: star cluster formation in a compact group environment. Astron. J..

[8] Williams BA, Yun MS, Verdes-Montenegro L (2002). The VLA H I observations of Stephan's Quintet (HCG 92). Astron. J.

[9] Xu C (2005). Ultraviolet emission and star formation in Stephan's Quintet. Astrophys. J..

[10] Trinchieri G (2005). Stephan's Quintet with XMM-Newton. Astron. Astrophs..

[11] Appleton PN (2006). Powerful high-velocity dispersion molecular hydrogen associated with an intergalacitic shock wave in Stephan’s Quintet. Astrophys. J..

[12] Duc P-A, Cuillandre J-C, Renaud F (2018). Revisiting Stephan's Quintet with deep optical images. Mon. Not. R. Astron. Soc. Lett..

[13] Shostak GS, Sullivan WT, Allen RJ (1984). H I synthesis observations of the high-redshift galaxies in Stephan's Quintet. Astron. Astrophys..

[14] Lisenfeld U (2002). Abundant molecular gas in the Intergalactic medium of Stephan’s Quintet. Astron. Astrophys..

[15] de Mello DF (2012). Star formation in HI tails: HCG 92, HCG 100 and six interacting systems. Mon. Not. R. Astron. Soc..

[16] Xu C, Sulentic JW, Tuffs R (1999). Starburst in the intragroup medium of Stephan's Quintet. Astrophys. J..

[17] Guillard P (2022). Extremely broad Lyα line emission from the molecular intragroup medium in Stephan's Quintet: evidence for a turbulent cascade in a highly clumpy multi-phase medium?. Astrophys. J..

[18] Renaud F, Appleton PN, Xu CK (2010). N-body simulation of the Stephan's Quintet. Astrophys. J..

[19] Hwang J-S (2012). Models of Stephan's Quintet: hydrodynamic constraints on the group's evolution. Mon. Not. R. Astron. Soc..

[20] Peterson SD, Shostak GS (1980). Stephan's Quintet: H I distribution at high redshifts. Astrophys. J..

[21] Borthakur S (2015). Distribution of faint atomic gas in Hickson compact groups. Astrophys. J..

[22] Beck R (2016). Photometric redshifts for the SDSS Data Release 12. Mon. Not. R. Astron. Soc..

[23] Schneider SE (1989). Multifrequency survey of the intergalactic cloud in the M96 group. Astron. J.

[24] Yun MS, Ho PTP, Lo KY (1994). A high-resolution image of atomic hydrogen in M81 group of galaxies. Nature.

[25] Koopmann R (2008). A 500 kpc HI extension of the Virgo pair NGC 4532/DDO 137 detected by the Arecibo Legacy FAST ALFA (ALFALFA) survey. Astrophys. J..

[26] Leisman L (2021). The ALFALFA almost-dark galaxy AGC 229101: a two billion solar mass HI cloud with a very low surface brightness optical counterpart. Astron. J.

[27] Duc P-A, Bournaud F (2008). Tidal debris from high-velocity collisions as fake galaxies: a numerical model of VIRGOHI 21. Astrophys. J..

[28] Michel-Dansac L (2010). A collisional origin for the Leo Ring. Astrophys. J..

[29] Corbelli E, Schneider SE, Salpeter EE (1989). HI mapping of outer disks of galaxies: M33 and NGC 3344. Astron. J.

[30] Field GB (1965). Thermal instability. Astrophys. J..

[31] Nelson D (2020). Resolving small-scale cold circumgalactic gas in TNG50. Mon. Not. R. Astron. Soc..

[32] Jiang P (2020). The fundamental performance of FAST with 19-beam receiver at L band. Res. Astron. Astrophys..

[33] Zhu M (2021). FAST discovery of a long HI accretion stream toward M106. Astrophys. J. Lett..

[34] Cheng C (2020). The atomic gas of star-forming galaxies at z ∼ 0.05 as revealed by the Five-hundred-meter Aperture Spherical Radio Telescope. Astron. Astrophys..

